# Prototype of Monitoring Transportation Pollution Spikes through the Internet of Things Edge Networks

**DOI:** 10.3390/s23218941

**Published:** 2023-11-03

**Authors:** Eric Nizeyimana, Damien Hanyurwimfura, Junseok Hwang, Jimmy Nsenga, Dereje Regassa

**Affiliations:** 1 Africa Centre of Excellence in Internet of Things (ACEIoT), University of Rwanda (UR), Kigali P.O. Box 3900, Rwanda; hadamfr@gmail.com (D.H.); jimmy.nsenga@gmail.com (J.N.); 2 Global R&D Center (GRC), Seoul National University, Seoul 08826, Republic of Korea; givealltoyou@gmail.com

**Keywords:** IoT edge networks, air pollution spikes, transportation, smart cities, real-time monitoring

## Abstract

Air pollution is a critical problem in densely populated urban areas, with traffic significantly contributing. To mitigate the adverse effects of air pollution on public health and the environment, there is a growing need for the real-time monitoring and detection of pollution spikes in transportation. This paper presents a novel approach to using Internet of Things (IoT) edge networks for the real-time detection of air pollution peaks in transportation, specifically designed for innovative city applications. The proposed system uses IoT sensors in buses, cabs, and private cars. These sensors are equipped with air quality monitoring capabilities, including the measurement of pollutants such as particulate matter (PM2.5 and PM10), nitrogen dioxide (NO2), ozone (O3), sulfur dioxide (SO2), and carbon dioxide (CO2). The sensors continuously collect air quality data and transmit them to edge devices within the transportation infrastructure. The data collected by these sensors are analyzed, and alerts are generated when pollution levels exceed predefined thresholds. By deploying this system within IoT edge networks, transportation authorities can promptly respond to pollution spikes, improving air quality, public health, and environmental sustainability. This paper details the sensor technology, data analysis methods, and the practical implementation of this innovative system, shedding light on its potential for addressing the pressing issue of transportation-related pollution. The proposed IoT edge network for real-time air pollution spike detection in transportation offers significant advantages, including low-latency data processing, scalability, and cost-effectiveness. By leveraging the power of edge computing and IoT technologies, smart cities can proactively monitor and manage air pollution, leading to healthier and more sustainable urban environments.

## 1. Introduction and Background

Air pollution is one of cities’ biggest environmental and public health challenges worldwide [1,2]. The adverse effects of air pollution on human health, ecosystems, and the climate are well-documented, making it a critical issue that requires immediate attention [2]. In urban areas, transportation is a significant contributor to air pollution, releasing various pollutants such as particulate matter (PM2.5 and PM10), nitrogen dioxide (NO2), ozone (O3), Sulfur dioxide (SO2), carbon dioxide (CO2), and carbon monoxide (CO [3]). This research deals with air pollution in transportation, especially in urban areas.

Air pollution is characterized as a silent but deadly menace, leading to an annual death toll of seven million individuals [4]. Alarmingly, approximately 90% of the global population is consistently exposed to its harmful effects [4]. Both long and short exposures are dangerous to our health [5]. The many diseases associated with air pollution include respiratory and cardiovascular diseases [6]. Air pollution exerts significant adverse effects on human health, manifesting in various forms, such as respiratory diseases, including asthma and lung cancer, as well as cardiovascular dysfunctions and malignancies [7]. Research has identified a strong correlation between male infertility and exposure to air pollution, and it has established a link between air contamination and an elevated risk of immune dysfunction, neuroinflammation, neurobehavioral hyperactivity, criminal behaviors, premature aging, Alzheimer’s disease, and Parkinson’s disease [7]. Notably, traffic-related air pollutants have been implicated in skin aging and the development of pigmented facial spots.

Furthermore, air pollution is associated with eye irritation, dry eye syndrome, retinopathy risk, and adverse ocular outcomes [7]. Chronic exposure to air pollutants during pregnancy is linked to negative effects on fetal development, including low birth weight and stillbirth.

Additionally, air pollution is recognized as a significant contributor to the increased prevalence of allergic diseases in children [7]. In implementing this project, the expectation is to decrease deaths from air pollution. The research elaborates on policies that can be adopted to enhance the well-being of individual car owners and the broader community, ultimately contributing to an increase in the overall life expectancy of the global population.

The adverse impacts of air pollution affect pregnant women, children, elderly adults, and individuals residing in rural areas [6]. Numerous studies have linked exposure to air pollutants with impaired brain development, learning disabilities, and children’s lower intelligence quotient (IQ) [8]. Children’s developing brains are more vulnerable to the harmful effects of air pollution than adults [8].

Here are some ways in which air pollution can impact the IQ of children:

- Neurodevelopmental effects: Exposure to air pollutants, especially fine particulate matter (PM2.5) and nitrogen dioxide (NO2), has been associated with neuroinflammation and oxidative stress in the brain [8,9,10,11]. These inflammatory responses can disrupt normal brain development, affecting cognitive functions and potentially leading to lower IQ scores [11].
- Cognitive decline: Long-term exposure to air pollution during childhood has been linked to cognitive decline later in life [12,13]. This decline may manifest as memory deficits, reduced attention span, and impaired decision-making abilities, all of which can influence IQ scores.
- Impact on academic performance: High levels of air pollution have been associated with reduced academic performance among children. Students exposed to chronic air pollution may experience difficulties learning and achieving academic milestones, affecting their IQ development.
- Structural brain changes: Studies have shown that exposure to air pollution can lead to structural changes in the brain, including alterations in the hippocampus and frontal cortex, areas crucial for learning, memory, and IQ development [14].
- Prenatal exposure: Prenatal exposure to air pollution can also significantly impact children’s cognitive development. Studies suggest that exposure to air pollutants during pregnancy can lead to lower IQ scores and increased risk of cognitive impairments in children [8].
- Socioeconomic disparities: Children from low-income communities often face higher levels of air pollution due to their proximity to industrial facilities and traffic congestion. As a result, they may experience more significant cognitive impairments, exacerbating existing socioeconomic disparities [15].

It is important to note that while there is a growing body of evidence linking air pollution to cognitive deficits in children, the specific mechanisms and causal relationships of this are still subjects of ongoing research. Furthermore, individual susceptibility can vary based on genetics, socioeconomic status, and preexisting health conditions [15,16]. Efforts to address this issue include advocating for stricter air quality standards, promoting cleaner transportation options, and raising awareness about the potential risks of air pollution, especially for vulnerable populations such as children [17,18]. Due to the adverse effects of air pollution, vulnerable individuals are disproportionately impacted. Children, the future of our society, are particularly susceptible to the adverse effects of air pollution, which can affect their cognitive development. As a result, it is imperative to implement measures to safeguard their health and well-being, and this research will do that.

In urban environments, the escalating issue of air pollution poses substantial risks to public health, with transportation systems being significant contributors. Air pollution spikes can harm children’s cognitive development and intelligence quotient (IQ) [10]. The rapid and accurate detection of air pollution spikes caused by vehicular emissions is imperative to protect citizens’ well-being [3]. However, the current monitoring methods often lack real-time capabilities and struggle to capture dynamic pollution fluctuations [19]. The emergence of Internet of Things (IoT) edge networks offers a promising avenue for establishing efficient, responsive, localized air quality monitoring systems. This research aims to harness IoT edge networks to detect real-time air pollution spikes in transportation, enabling timely interventions and safeguarding public health [20]. This research proposes a system capable of capturing spikes in pollution from transportation, assessing their impact on health, and suggesting methods to mitigate these effects.

Measuring spikes in air pollution from transportation is paramount due to its significant implications for public health and the environment. Air pollution spikes have a health impact on vulnerable populations such as children, the elderly, pregnant women, and individuals with preexisting health conditions who are particularly susceptible to the health effects of air pollution. Short-term spikes in air pollution can trigger respiratory and cardiovascular issues, exacerbating conditions such as asthma, bronchitis, and heart disease [21]. Increased pollution levels often lead to a surge in hospital admissions and emergency room visits, placing a strain on healthcare systems [22]. With this prototype system, the levels of air pollution that can impact vulnerable individuals will be continuously monitored. When these levels exceed a predetermined threshold, notifications will be sent to the relevant authorities.

Pollution spikes from transportation sources can occur suddenly due to traffic congestion or meteorological conditions, resulting in immediate health risks [23,24]. Even brief exposure to elevated pollution levels can lead to adverse health effects. Repeated exposure to pollution spikes can contribute to chronic health problems, including reduced lung function and long-term cardiovascular issues [25]. Ecosystems are in danger of being damaged due to air pollutants, which can harm plants, water bodies, and soil, impacting biodiversity and ecosystem health. Some pollutants can lead to the formation of acid rain, which harms aquatic life and damages buildings and infrastructure [11]. This research introduces a developed prototype system for monitoring air pollution resulting from transportation, specifically from cars. When any spikes in pollution occur, the system will automatically trigger an analysis by the relevant authorities to assess its impact. Consequently, this proposed system holds the potential to enhance both public health and environmental quality by effectively reducing air pollution originating from transportation, particularly in urban areas.

Black carbon, a component of particulate matter emitted from transportation, contributes to climate change by absorbing sunlight and accelerating ice melting in polar regions [26]. Air pollution spikes can lead to violations of air quality standards set by regulatory bodies, necessitating corrective measures to ensure compliance. The real-time monitoring of spikes informs the development and implementation of effective pollution control policies and traffic management strategies [27]. Providing real-time information about pollution spikes empowers individuals to take protective actions, such as reducing outdoor activities or adjusting travel plans [23,24]. Accessible air quality data encourage public advocacy for cleaner transportation options and policies [28]. This research aims to inform the development of policies that can provide real-time solutions for deviations in transportation based on the specific locations of pollutants.

Some IoT edge network systems were proposed by previous researchers to monitor air pollution [29,30,31]. The paper [28] proposed a framework for designing air pollution using IoT edge networks, blockchains, and artificial intelligence. Most of these proposed systems are only for monitoring air pollution in general; they are not specific to transportation spike pollutants. In the current research, this paper focuses on vehicle air pollution.

Monitoring spikes helps authorities identify pollution hotspots and target interventions where they are most needed [32]. Precise data allow for the efficient allocation of resources for pollution mitigation efforts. Lastly, studying pollution spikes contributes to a better understanding of air quality dynamics, pollutant behavior, and their impacts. Data on pollution spikes can be used in educational campaigns to inform people about the importance of reducing pollution sources [33]. Monitoring spikes in air pollution from transportation sources is critical for safeguarding public health, protecting the environment, and fostering informed decision making at individual and policy levels [20]. Timely action to mitigate these spikes can lead to improved air quality, enhanced public well-being, and a more sustainable future [17].

Based on the analysis of existing systems, it becomes evident that this paper introduces a novel system poised to play a pivotal role in combatting transportation-related pollution. There are dynamic pollution patterns where air pollution levels can vary widely across time and locations due to traffic density, weather conditions, and industrial activities. Existing monitoring approaches may miss rapid pollution spikes, delaying necessary actions. The need for real-time data can prompt the identification of pollution spikes, which demands real-time data collection and processing. Traditional centralized monitoring systems face latency issues, hindering timely responses. There should be spatial variability as the air quality can significantly differ even within short distances due to local sources of pollution. Establishing localized monitoring to capture these variations is a challenge. Edge network reliability is still challenging in IoT edge networks since they rely on distributed sensors deployed on vehicles or infrastructure. Ensuring sensor accuracy, reliability, and data synchronization in these dynamic networks is crucial. Integrating data from various sensors, vehicle fleets, and stationary sources necessitates robust data fusion and analytics techniques to identify pollution spikes accurately. The last challenge we examined in this paper was privacy and security concerns for collecting real-time data from transportation sources. Designing systems that respect individuals’ privacy while providing valuable insights is essential.

## 2. Literature Review

This section serves as an introduction to existing systems relevant to the development of the system in this research. It aims to provide an overview of these systems, highlighting their respective strengths and weaknesses compared to the developed system. Furthermore, it will compare various research aspects between the related systems and the one set in this study.

In their paper [34], the authors proposed a novel approach for designing a cost-effective and real-time air pollution monitoring system by leveraging edge computing and Internet of Things (IoT) technologies. Current air quality monitoring systems often lack the necessary spatial and temporal resolutions, posing challenges to accurately assessing air pollution. The proposed system employs sensors to collect real-time air quality data to address these issues, which is then transmitted to edge computing devices for processing and analysis [35]. This approach reduces the computational load on battery-powered sensing nodes while maintaining accuracy. The paper overviews existing monitoring systems, their limitations, challenges, and the proposed edge computing-based IoT architecture for air quality monitoring [35]. From an analysis of these two papers, this research demonstrates the feasibility of applying edge IoT with real-time systems to address air pollution. The approach proposed in these papers has been incorporated into this research to be applied within the realm of transportation, particularly for managing spikes in air pollution.

The study on developing an artificial intelligence (AI) model to measure traffic-related air pollution using multisensory and weather data investigates the impact of various input variables on training air quality indexes through fuzzy logic combined with simulated annealing (SA) and particle swarm optimization (PSO) [36]. The model predicts concentrations of NO2 and CO based on resistivity values from multisensory devices and weather variables. PSO outperforms SA in the optimization process, and the study highlights the sensitivity of input resistivities for predicting NO2 and CO concentrations, which is crucial for addressing air pollution challenges. One paper [36] focused on only two specific pollutants. However, this research developed a prototype to address six significant pollutants that significantly impact air quality worldwide.

One paper [37] introduced portable IoT air quality monitoring devices for vehicle installation in Ibarra City, Ecuador. These devices use Message Queuing Telemetry Transport (MQTT) to send data to a time series database in edge computing and cloud computing for visualization. The research employs outlier detection and supervised classification to analyze data, determining air pollution levels categorized as low, normal, and high. The results show over 90% performance for IoT nodes inferring air quality, with a memory consumption of 14 Kbytes in flash and 3 Kbytes in Random Access Memory (RAM). This approach presents an efficient, cost-effective solution for air quality measurement through IoT technology. However, it is important to note that the system, in its current form, does not measure air pollution directly from the vehicle itself. In the proposed prototype developed within this research, the system will be integrated into individual vehicles, allowing for the measurement of pollutants emitted by each car over time to ensure their continued compliance with air quality standards. If a vehicle exceeds the established pollution thresholds, specific policies and measures will be implemented accordingly.

One paper [38] discussed the integration of IoT, edge intelligence, 5G, and blockchain technologies into autonomous vehicles (AVs) to enhance their efficiency and sustainability. AVs are becoming a vital part of intelligent transportation systems. The study reviews the impact and implementation of these technologies, addressing challenges and providing insights into their seamless integration. This integration aims to provide self-verifying, self-executing, and secure AV systems. This current research is based on some challenges defined in the paper [38] and addresses solutions accordingly.

The study from [39] investigates the potential application areas of low-cost PM (particulate matter) sensors for air quality monitoring. The focus is on evaluating the performance of two PM sensor models, PMS5003 and SPS30, based on the US Environmental Protection Agency (EPA) guidelines. The sensors are assessed in terms of indicators such as the coefficient of determination (R2), root mean squared error (RMSE), mean absolute error (MAE), mean normalized bias (MNB), and coefficient of variation (CV). The aim is to determine if these sensors could be used as supplemental monitoring tools to enhance existing air quality measurement networks. The study contributes to understanding the effectiveness of low-cost sensors for air quality assessment. The previous system only focused on monitoring a single pollutant, PM. However, in the current research, a low-cost monitoring system has been developed to track six different pollutants simultaneously.

Another paper [40] focused on improving indoor air quality (IAQ) monitoring systems. It addresses the need to balance well-being and energy efficiency in building design, especially given the impact of indoor air quality on occupants’ health and comfort. The authors propose an innovative approach that considers pollutant concentrations and exposure time, developing new indices. They integrate sensors and IoT technology to create a monitoring system that can autonomously or remotely control ventilation based on IAQ data and predefined thresholds. The study introduces decision-making algorithms to account for measurement uncertainty and validate the approach using simulated data. The current research leverages IoT edge networks and an algorithm to monitor outdoor air pollution from vehicles while considering spikes.

The authors of paper [41] introduced an adaptive hardware–software platform for predictive air quality monitoring, explicitly focusing on carbon dioxide (CO2) concentrations. The aim was to forecast CO2 trends accurately to prevent sudden increases and enable heating, ventilation, and air conditioning (HVAC) systems to control indoor conditions effectively while avoiding energy waste. The system employs deep learning algorithms to analyze a limited window of recent data, making it adaptable to changing habits and environmental conditions. The platform achieves high accuracy, mainly using the long short-term memory network. This innovative approach addresses IAQ concerns, especially with increased indoor time due to factors such as COVID-19. The current research employs a similar approach to test the developed prototype within a laboratory setting but with a notable difference: it assesses the system’s performance for six different pollutants.

Another paper [42] discussed the significance of air quality monitoring and control in the context of smart cities. It presents a detailed framework for air pollution monitoring and prediction using the Internet of Things (IoT) architecture. Moreover, paper [43] examined low-cost air quality monitoring devices to assess road-transport-related emissions, aiming to supplement high-precision monitoring stations. A case study conducted in Munich focuses on PM10 measurements. Ten low-cost devices were deployed to gather hourly PM10 values. A statistical analysis of historical data revealed correlations between PM10 concentrations, weather conditions, and traffic volumes. The study found that some expected relationships were evident in the data from low-cost devices, but others were inconclusive. The research highlights the importance of extended measurement periods, frequent calibration, and the challenges of interpreting point measurements. It suggests that dispersion models can aid in understanding complex factors affecting air quality. The current research delves deeper into the proposed framework, particularly in calibrating all six pollutants for application in transportation.

One paper [44] introduced a modular IoT sensing platform with hybrid learning capabilities for air quality prediction. A team of researchers from various institutions in India conducted the study. The focus is on addressing the challenges of accurate and low-cost air quality measurements in underdeveloped countries. The proposed platform includes an IoT node with multiple sensors to monitor pollutants such as ammonia (NH3), NO2, CO, and PM2.5, along with air humidity and ambient temperature. The system uses Global System for Mobile Communication (GSM)/WiFi technology to transmit real-time air quality data, generate alerts, and facilitate data analyses for environmental intelligence applications. The goal was to enhance the field of distributed, low-cost sensing devices for environmental monitoring. The study emphasizes the need for precise measurements and predictions of pollutants to combat the increasing ecological issues caused by urbanization and fossil fuel usage. However, the current research can capture spikes from vehicles in urban areas and subsequently use policies to take action. Furthermore, the prototype proposed in this research encompasses a broader range of pollutants than the previous one.

Another paper [45] introduced a framework for air pollution monitoring in smart cities using IoT and smart sensors. Authored by Arshad Ali, the study addresses the challenges of urbanization and the increasing city population, leading to heightened pollution levels. The proposed solution involves IoT and intelligent sensors continuously monitoring various environmental parameters such as humidity, carbon emissions, temperature, smoke, and hazardous particulates. The collected data are transmitted to a central office for analyses and action, contributing to improving the city’s environment. The current prototype solved the issues presented in this paper, which come from the population increase in cities.

The authors of the paper [46] addressed the increasing challenges of urbanization and pollution by leveraging the IoT paradigm and smart sensors. The framework aims to monitor various pollutants using a network of static and mobile sensors integrated through gateways. Data are transmitted wirelessly to the cloud, undergoing processing and analyses. The architecture accounts for sensor defects, power consumption, and data management. The study presents an overview of current trends and introduces a comprehensive cloud-centric architecture for efficient air quality monitoring in urban environments. The current research is now focusing on vehicle pollutants and capturing spikes.

Article [47] discussed using artificial intelligence (AI) in air quality monitoring for smart city management. The authors, En Xin Neo et al., present a comprehensive study that utilizes machine learning and deep learning techniques to predict air quality. They propose an end-to-end predictive model incorporating various pollution markers and meteorological data for four different urban cities in Selangor, Malaysia. The study highlights the importance of feature optimization to enhance the accuracy of air quality predictions, particularly for PM2.5 concentration. While this paper exclusively focused on a single pollutant from a general perspective, the current research specifically addresses vehicles’ spikes.

The study from paper [48] introduced a vehicle sensor network (VSN) approach for monitoring air quality in urban areas using low-cost IoT devices mounted on vehicles. The devices collect geolocated particulate matter (PM) measurements that are transmitted to an IT infrastructure. Real-time spatial and temporal pollutant distribution maps are generated and made accessible online. The VSN system was deployed in Trieste, Italy, with support from volunteers and local transportation authorities. The results reveal areas with poor air quality linked to increased vehicular traffic. The VSN approach offers valuable insights for urban planning and encourages reduced private car usage in favor of public transportation, enhancing air quality.

This research has the potential objective of harnessing IoT edge networks for real-time air pollution spike detection in transportation to safeguard public health. This research develops a distributed sensor network that establishes a network of IoT sensors deployed on vehicles and at strategic locations along transportation routes to capture real-time air quality data. This research implements edge computing capabilities to process sensor data locally, enabling the rapid detection of air pollution spikes without significant latency. In this paper, algorithms to analyze data patterns and identify sudden increases in pollutant levels that characterize pollution spikes were designed. This paper proposes policies to collaborate with governmental agencies responsible for environmental protection and public health to ensure data integration into decision-making processes and generate evidence-based recommendations for policy changes and regulatory measures to reduce transportation-related air pollution. This research uses machine learning techniques to develop predictive models for anticipating pollution spikes based on historical data and environmental conditions. This research contributes to the scientific community by sharing insights, data, and methodologies that can advance the understanding of air pollution’s impact on health and the effectiveness of IoT-based solutions.

Table 1 below provides a summary of the existing systems and the current prototype developed. It highlights all the unresolved challenges in the previous designs and outlines the solutions implemented by the current research.

In this table, we aim to compare the previous systems and the innovations introduced by the current research, addressing previously unmet challenges.

In conclusion, the global challenge of urban air pollution’s impact on health and the environment, with a focus on transportation, is a major contributor to harmful pollutants. Children’s cognitive development, often neglected, is highlighted as vulnerable to air pollution’s detrimental effects, with studies showing links to lowered IQ scores and learning disabilities. Rapid pollution spikes from transportation pose urgent risks, particularly for vulnerable groups, emphasizing the need for real-time detection and intervention. Leveraging IoT edge networks emerges as a solution to revolutionize air quality monitoring, necessitating collaboration, accurate sensors, and privacy safeguards. In the next section, the paper highlights the materials and methodology used to achieve the results required.

## 3. Materials and Methods

This section explains the materials and methodology used in this research. The presented study aims to detect air pollution peaks in transportation using real-time IoT edge networks. The prototype was developed in the laboratory of Seoul National University and includes a comprehensive setup of materials and a well-defined methodology. Sensors such as high-precision air quality sensors were used as materials to measure various pollutants such as particulate matter (PM2.5 and PM10), carbon dioxide (CO), ozone (O3), sulfur dioxide (SO2), and nitrogen dioxide (NO2). For prototyping, IoT-enabled microcontrollers, such as Arduino UNO, were used to interface with the sensors for local data processing and communication with edge networks. ESP8266 WiFi wireless communication modules enabled data transfer between edge devices and central systems. Local processing functions allow for the preprocessing of data and rapid identification of pollution spikes before relevant information is transmitted using a spike detection algorithm. A central repository was set up to collect and store the transmitted data for further analyses and visualization. The real-time air pollution spike detection method involves a multi-step process that takes advantage of edge computing and IoT technologies, as explained in this section [49]. The paper proposes to generate and share real-time alerts and reports with relevant stakeholders, including local authorities, health departments, and the public.

### 3.1. Hardware Components

The prototype system comprises a microcontroller, sensors, and communication and transmission devices. These hardware devices are connected to capture the air pollution from different sensors on different cars that are in transit.

**Figure 1:**
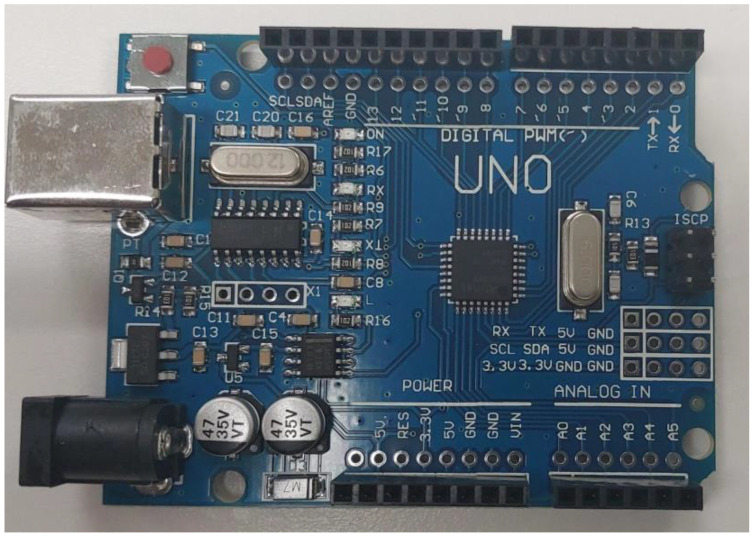
Arduino UNO board.

From Figure 1, the ATmega328P is the core of Arduino Uno, with a 32 KB flash memory for code storage, 2 KB SRAM for data, and 1 KB EEPROM for non-volatile storage. It offers digital I/O pins for interfacing with sensors and actuators, six analog input pins, timers for precise timing, and PWM for tasks such as motor control. Communication is possible through UART, I2C, and SPI interfaces, supporting various clock frequencies. This microcontroller is the brain behind Arduino projects, enabling code control of connected components.

The system comprises different types of sensors from other manufacturers with high precision. All these sensors have threshold values for good air quality as defined by the World Health Organization (WHO), as shown in Table 2.

Table 3 provides a comprehensive overview of all the sensors utilized in this research, offering detailed descriptions of each sensor’s functionality and purpose. The table also includes visual representations of these sensors, which can measure specific environmental pollutants.

**Table 3:** Sensors description.

Serial Number	Sensor Name	Description
1	Particulate Matter sensor (PM2.5 and PM10)	PM sensors measure fine airborne particles, such as PM10 and PM2.5, impacting air quality and health. They employ optical scattering and absorption principles, including a light source, sample chamber, detector, and signal processing, which are crucial for monitoring respiratory and cardiovascular risks. The research incorporates the Plantower PMS5003 sensor, offering real-time, precise particle concentration data via a digital interface, as shown in Figure 2.
2	Temperature and Humidity Sensor	A temperature and humidity sensor, or hygrothermometer, measures ambient temperature and relative humidity. These sensors are vital for weather monitoring and indoor climate control, utilizing capacitance or electrical resistance changes to gauge humidity and providing temperature and humidity readings in Celsius or Fahrenheit and percentage relative humidity (% RH). Figure 3 illustrates the specific temperature and humidity sensor utilized in the project.
3	CO2 sensor	DFRobot’s CO2 sensor utilizes voltage output to detect CO2 levels, with a customizable threshold for digital signal activation. It employs an MG-811 gas sensor, offering CO2 sensitivity and stable performance, while its industrial-grade design ensures reliability. Calibration is necessary, operating at 5 V, with compatibility for the Gravity interface, and featuring a compact 32 × 42 mm size. The sensor is shown in Figure 4.
4	Ozone Sensor	In this research, the MQ131 O3 sensor is employed to measure ozone (O3) gas concentration, which is essential for Earth’s stratosphere but harmful at ground level. Figure 5 depicts the MQ131 sensor used in the project, which operates through chemo-resistive gas sensing, detecting ozone-induced resistance changes in its sensitive material. The sensor includes a heater for temperature control and signal conditioning circuitry, facilitating ozone concentration determination through output signal processing. The sensor is shown in Figure 5.
5	SO2 Sensor	SO2 sensors are pivotal in monitoring environmental air quality and health by detecting sulfur dioxide (SO2) gas concentrations. They utilize diverse technologies such as electrochemical, optical, and chemical absorbance methods, each offering distinct detection mechanisms while outputting visual changes that correlate with SO2 concentration. Figure 6 illustrates the SO2 sensor employed in this research project.
6	NO2 Sensor	Nitrogen dioxide (NO2) sensors are designed to measure the concentration of this harmful gas generated from combustion processes in vehicles, power plants, and industry. They employ surface adsorption, detecting changes in properties such as electrical conductivity or mass to quantify NO2 levels. These sensors find applications in vehicles for emissions monitoring and engine optimization due to their critical role in health and environmental assessments. Figure 7 illustrates the NO2 sensor.
7	ESP8266 Wifi module	The ESP8266 has been a driving force behind the proliferation of IoT projects and applications due to its affordability, capabilities, and ease of integration. The WiFi module supports data transmission. It only transmits once the value exceeds the threshold. Figure 8 shows the module.

### 3.2. Hardware Architecture

Figure 9 shows the designed architecture that combines all hardware components explained above.

Figure 9, an image of the architecture, shows how all hardware components are connected to capture the air pollution from the environment on cars and where the cars are passing.

Below is Figure 10, displaying the prototype meticulously designed with all sensors seamlessly integrated onto an Arduino Uno board. This innovative prototype can detect all pollutants commonly associated with vehicles, as elucidated in this paper. Moreover, the system is seamlessly integrated into the car’s power distribution system, enabling it to initiate automatically when the vehicle is powered on. As depicted in Figure 10, the system is securely enclosed within a protective box, safeguarding it from potential damage while preserving the necessary air capture space from the car’s exhaust to which it is affixed.

### 3.3. Mathematical Tools

This paper uses mathematical concepts to introduce a novel approach to capturing spikes in air pollution data. We identify spikes in the sensor data by employing the Heaviside step function and Dirac delta function on data corrected by a specially designed prototype, as shown in the figure. This process involves applying these mathematical functions to the sensor data and calculating the spike function.

We apply both the Heaviside step function and the Dirac delta function from each sensor’s dataset. The application of the Heaviside step function serves to emphasize sudden changes or abrupt increases in pollution levels. Simultaneously, the Dirac delta function helps pinpoint specific time points where pollution levels experience instantaneous spikes.

Two essential mathematical functions are pivotal in this analysis: the Heaviside step function and the Dirac delta function. The Heaviside step function, denoted as H(t), is used to model the sudden onset of a phenomenon. The Dirac delta function, denoted as δ(t), represents an infinitely narrow impulse at a specific point.

The Heaviside step function is

(1) $$V\left(x\right)=H\left(x-ti\right)\;\;\;\;\;\;\;\;\;\;\;\;\;\;\;\;x\geq ϴ$$

where V(x) is the input signal of the sensor data at time x. Then, H(x−ti) is the Heaviside step function at time x and is great or equal to the threshold, ϴ. That means the function gives 0 when the signal from the sensor is less than the threshold, ϴ. Then, once the value at time x is greater than the threshold, ϴ, the value becomes 1. ti denotes the peak at a specific point.

The Dirac delta function, denoted as δ(x), is a distribution or generalized function used to represent point sources or impulses.

(2) $$\delta (x)=\left\{\begin{array}{c} 0,\;\;\;\;\;\;\;\;\;\;\;for\;x\;\neq 0\\ \infty ,\;\;\;\;\;\;\;\;\;\;\;\;for\;x=0 \end{array}\right.$$

δ(x) has an unusual property where its integral over the entire real line equals 1.

(3) $$\int_{-\infty }^{+\infty }\delta \left(x\right)dx=1$$

Then, by applying Equation (3) to the Heaviside function (1), we obtain the peak value at time (x−ti). The Dirac delta function ensures that the spike is localized at ti. Then, the peak function will be Equation (1) multiplied by Equation (3):

(4) $$Peak\left(x-ti\right)=H\left(x-ti\right)\times \delta (x-ti)\;\;\;$$

We calculate the spike function for each sensor dataset based on applying the Heaviside step function and the Dirac delta function. The spike function quantitatively represents the detected spikes, providing insight into the intensity and duration of pollution events.

The spike function will be measured based on the height of the peak, H, at a specific point, ti, for a value greater than or equal to the threshold, θ. Then, the spike function from Equation (4) can be denoted as:

(5) $$Spike\left(x\right)=H\times Peak\left(x-ti\right)\;\;for\;x\geq ϴ$$

Then, from Equation (5), let us analyze spike behaviors when data come from the sensors by applying sigmoid functions. Therefore, that function will give the continuous doted function from different points calculated in Equation (5). The sigmoid function is a function that creates a gradual rise in the sensor data when they approaches the threshold. Let us assume a sensor measures a physical quantity (e.g., temperature, NO2,…), then makes a model of spikes in the data whenever they goes above a certain threshold, θ. The combination of the sigmoid function and a scaling factor can be used to achieve this.

The sigmoid function is often defined as:

(6) $$S\left(x\right)=\frac{1}{1+e^{-x}}$$

To control the steepness of the rise near the threshold, you can multiply the input by a scaling factor, “*k*”:

(7) $$ScaledS\left(x\right)=\frac{1}{1+e^{\left(-k\times \left(x-ϴ\right)\right)}}$$

Then, applying Equation (7) to the height H, the result can be scaled to obtain scaled spikes in the below equation:

(8) $$SpikesSensorData(x)=\left\{\begin{array}{c} 0,\;\;\;\;\;\;\;\;\;\;\;\;\;\;\;\;\;\;\;\;\;\;\;\;\;\;\;\;\;\;\;\;\;\;\;for\;x<0\\ H*Scaled(x),\;\;\;\;\;\;\;\;\;\;\;\;\;\;for\;x\geq ϴ \end{array}\right.$$

In this function, the sensor data remain low for values below the threshold, θ. As the input, x, crosses θ, the sigmoid function causes the data to rise gradually, and then the spike is introduced by scaling the sigmoid function by the magnitude, H. The parameters θ, H, and k can be adjusted to control the function’s behavior.

This paper applies these mathematical functions as tools to analyze behaviors of spikes during the data capturing from the air pollution sensors. Some software components have been used to compute the algorithm from the mathematical model and the hardware components.

### 3.4. Software Components

This research has used different types of software to analyze the data captured by the developed prototype in Figure 10 and to implement the algorithm generated from the mathematical models explained in the previous subsection.

This research harnesses the Arduino IDE to facilitate the programming and control of an Arduino Uno microcontroller. The program uploaded to the Arduino Uno is designed to oversee the operation of multiple sensors, ensuring they operate according to their respective calibrations and measurement parameters. Subsequently, the acquired sensor data are transmitted to cloud servers using the server’s designated IP address for data transfer.

In addition to the Arduino IDE, this research also leverages the Python 3.7 programming language. Python is employed to conduct an in-depth analysis of the spike behaviors generated by each sensor integrated into the system. By combining these software tools into the research framework, a comprehensive approach is taken to collect, process, and analyze data, thereby facilitating a thorough investigation of the research objectives.

### 3.5. Algorithm for Spiking Detection

In one paper, the authors developed an algorithm specifically designed to address the critical issue of spike detection within air pollution monitoring systems deployed in transportation networks. The overarching objective of this algorithm is to contribute to improving public health in densely populated urban areas by identifying and characterizing elevated air pollution levels, often caused by factors such as vehicular emissions and industrial activities. As depicted in the accompanying flowchart, the algorithm represents a systematic approach to data analysis and pattern recognition, enabling the timely and accurate identification of pollution spikes. This research endeavors to detect spikes and provide valuable insights for developing proactive pollution control strategies and mitigation measures, ultimately fostering healthier urban environments and enhancing the well-being of the communities residing therein.

**Chart 1:**
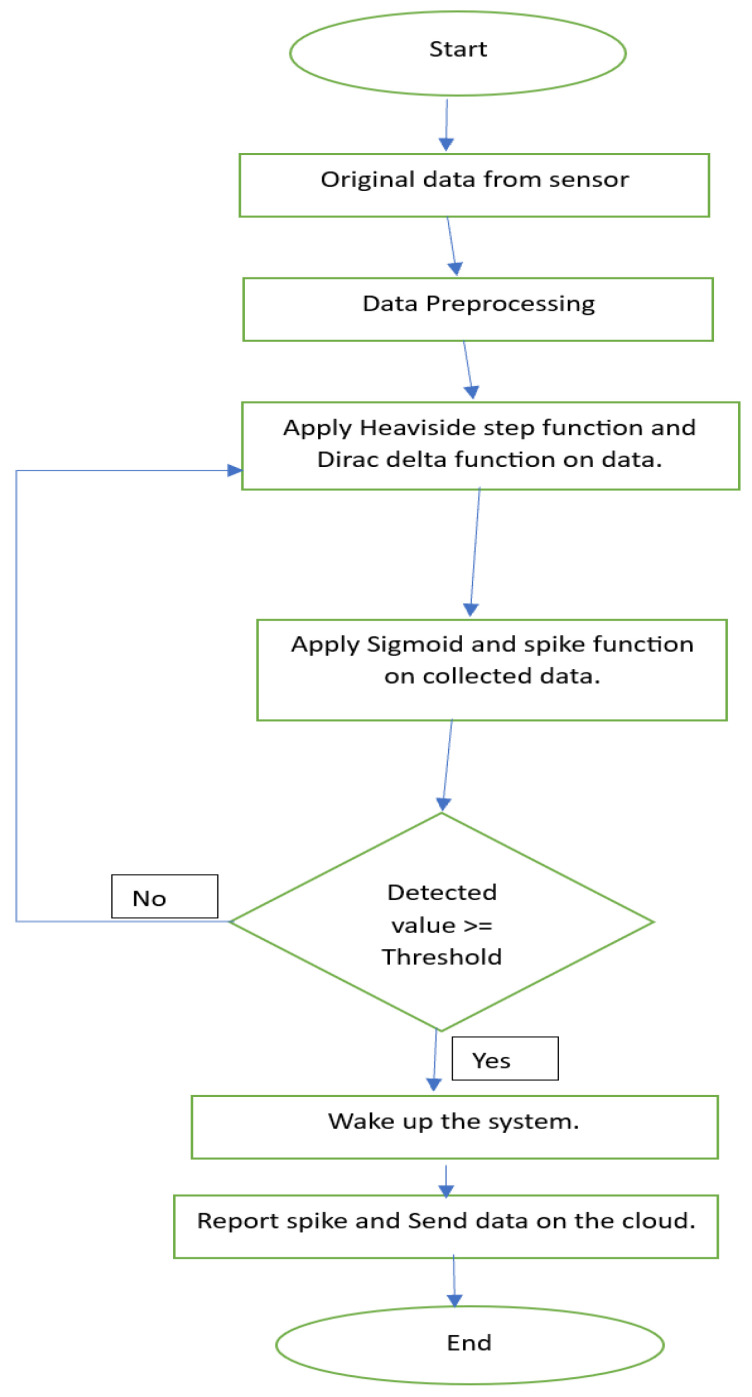
Flowchart for spikes algorithm.

Chart 1 is for the flowchart of the algorithm. The process commences with the sensors generating data from the surrounding environment. These data are meticulously collected and subsequently stored, preparing them for the preprocessing stage. The system employs the Heaviside step function and the Dirac delta function to ensure the accurate capture of spikes within the data. Following this step, a sigmoid function is applied to the discrete data points, transforming them into a continuous signal. Finally, the system proceeds to generate the spike function. The system will analyze the spike and determine if it is greater than the defined threshold of the sensor, as described in Table 1.

Algorithm 1 provides a high-level overview of the process involved in air pollution spike detection.
**Algorithm 1**. for air pollution spikes detection in transportation for better health  **Input:** Sensor data (Particulate matter levels, Temperature, Humidity, O3, NO2, SO2, CO2) **Output:** Pollution level, alerts for spikes or dangerous levels 
*Step 1: Data Collection*
Set up air quality monitoring sensors at predetermined locations.Continuously collect sensor data, including readings for various pollutants such as Temperature, Humidity, PM2.5, PM10, CO2, NO2, O3, and SO2.Record the timestamp for each data point.
*Step 2: Data Preprocessing*
Check for sensor anomalies and calibration issues.Handle missing or erroneous data through interpolation or data imputation techniques.Smooth the data to reduce noise using filters or moving averages.
*Step 3: Spike Detection*
Define a threshold for each pollutant.Compare the current data by applying the Heaviside step, Direc delta, Sigmoid, and Spike functions with the threshold values.If any parameter exceeds the threshold, generate an alert indicating a pollution spike.
*Step 4: Data Reporting and Visualization*
Display real-time or periodic pollution data on a dashboard.Provide historical pollution data for analysis and comparison.
*Step 5: Mitigation and Response*
Implement strategies to reduce pollution sources if persistent high pollution levels are detected.
*Step 6: Data Storage*
Store collected data in a secure and accessible database for future analysis and research.
*Step 7: Continuous Monitoring*
Continuously run the algorithm, repeating steps 1 to 5, to ensure ongoing air quality monitoring.

This research section explains the materials and methodology used to detect air pollution peaks in transport using real-time IoT edge networks. It describes high-precision air quality sensors used to measure various pollutants and IoT-enabled microcontrollers for data processing and communication with edge networks. Mathematical tools such as the Heaviside step function and the Dirac delta function are used to identify pollution peaks, and a software framework using Arduino IDE and Python is used for the data analysis. Furthermore, the study introduces a comprehensive algorithm for detecting air pollution spikes, which can significantly contribute to enhancing public health in urban regions through the identification and characterization of heightened pollution levels. This algorithm includes data acquisition, preprocessing, spike detection, reporting, and continuous monitoring.

## 4. Experimental Evaluation and Results

This section deals with a comprehensive examination of the research findings. It contains a detailed account of the results obtained from using each of the materials and methods explained in the previous section. Currently, the prototype is being tested in a controlled laboratory environment where the temperature and humidity values remain relatively stable, mainly due to the active operation of an air conditioning system during daytime hours. Nevertheless, spikes in sensor readings have been observed due to external environmental factors. In this section, the authors present the results of the newly developed prototype and explain the application of an algorithm to identify and categorize these spikes across the different sensors integrated into the system.

### 4.1. Sensor Caption Data for all Sensor

The sensors collect data from the physical environment. If the data fall below the predefined threshold, the system will not transmit the data to the cloud. Conversely, when the data exceed the predefined threshold, the system will send the data to the cloud. Figure 11, Figure 12 and Figure 13 display information from all the sensors on the server.

All the below figures (Figure 11, Figure 12 and Figure 13) illustrate the data collected from various sensors. These sensors are connected to a single Arduino board, enabling the system to transmit relevant data based on pollutant levels exceeding predefined thresholds from the vehicle’s exhaust port, where the system is installed. As a result, the system can now conserve energy, aligning with the proposal presented in the paper. Furthermore, it facilitates a broad spectrum of monitoring capabilities by situating measurement sensors at crucial locations where vehicles operate, thereby providing real-time pollutant concentration data. Moreover, by individually placing measurement sensors within cars, users can promptly assess the pollutant concentrations generated within the vehicle’s environment. This feature allows for swift actions to minimize pollutant emissions. Additionally, individually locating measurement sensors in vehicles can be a policy tool for users to reduce soot pollutant emissions during vehicle operation actively. Furthermore, this research has the potential to contribute to effective decision making and proactive pollution control strategies by integrating with existing transportation infrastructure and pollution monitoring systems.

As show Figure 11a, the data emanating from the temperature sensors present a remarkably consistent profile, marked by minimal fluctuations. This constancy can be attributed to the controlled laboratory environment, meticulously maintained at a steady temperature of 25 degrees Celsius, courtesy of an active air conditioning system. Under these controlled conditions, the temperature readings remain virtually constant, reflecting the precision and stability of the laboratory setting. However, it is essential to note that occasional variations do manifest, typically on days when the air conditioner (AC) is temporarily deactivated. During these interludes, the temperature levels may exhibit minor deviations, which can have a cascading effect on the other sensors in the system. Such variations are particularly significant in the context of air pollution monitoring, as they can potentially catalyze an increase in pollution levels. The interconnectedness of these sensor readings highlights the importance of a stable temperature environment to ensure the accuracy and reliability of the pollution data collected.

Figure 11b shows that the data derived from the humidity sensor portray a dynamic profile characterized by noticeable fluctuations in response to the operational status of the air conditioning (AC) system. When the AC is inactive or temporarily turned off, the humidity levels show a discernible uptick. Conversely, when the AC is engaged, the humidity levels register a decrease. These fluctuations in the humidity readings provide valuable insights into the interplay between environmental conditions and the operation of the air conditioning system. The data underscore the direct impact of AC systems on local humidity levels, a factor that must be considered when interpreting air pollution data. Importantly, it is crucial to acknowledge that the system is slated for deployment in an outdoor environment, where temperature and humidity are not subject to the controlled conditions imposed by an AC system. Consequently, ensuring suitable and consistent environmental conditions will be of paramount importance. To ensure the accuracy and reliability of the data, it is imperative to implement measures aimed at mitigating the potential influence of uncontrolled humidity fluctuations that may manifest in outdoor environments.

**Figure 8:**
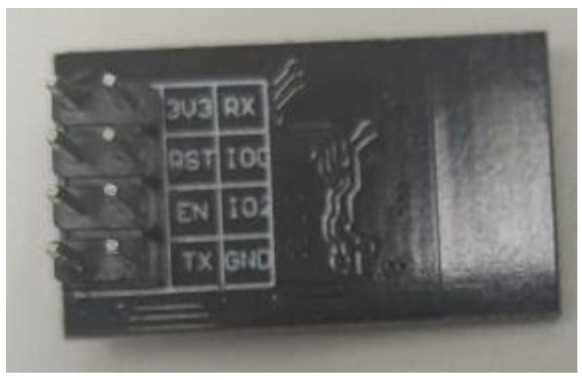
ESP8266 WiFi module.

**Figure 9:**
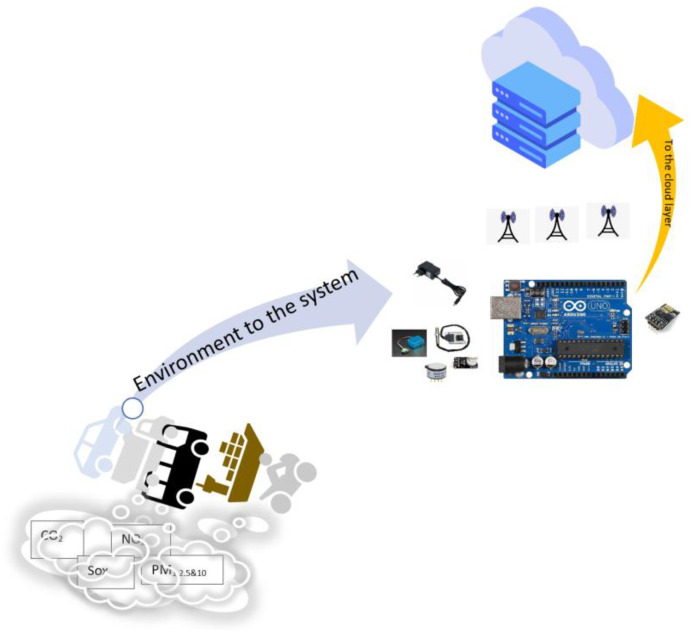
The prototype architecture of the developed system.

**Figure 10:**
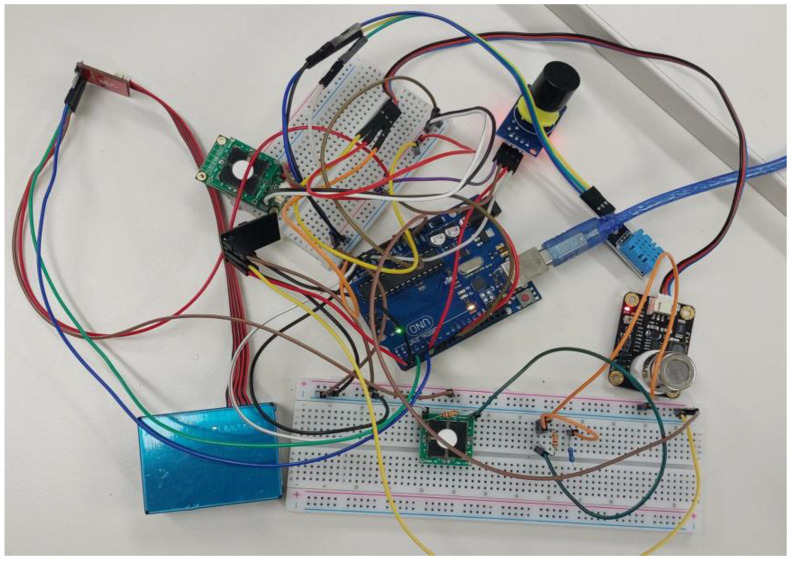
Prototype designed.

**Figure 11:**
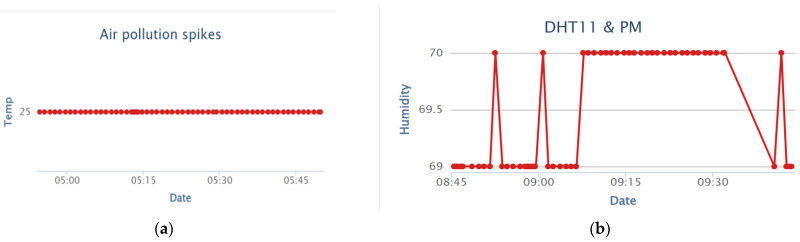
(**a**) Data on the server for temperature. (**b**) Data on the server for humidity.

**Figure 12:**
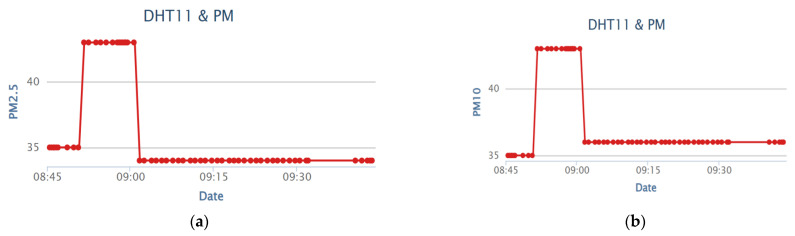
(**a**) PM2.5 data from the server. (**b**) PM10 data from the server.

Moving to Figure 12a, the dataset derived from the PM2.5 sensor reveals a crucial aspect of air quality monitoring. It encapsulates a range of particulate matter concentrations, specifically within the spectrum of 35 to 70 micrograms per cubic meter. This range is significant because it aligns with the air quality standards established by the World Health Organization (WHO), which defines these threshold values as indicative of acceptable air quality conditions. An intricate system operation comes into play as the sensor data are processed in real-time. Data points falling below the lower threshold of 35 micrograms per cubic meter or exceeding the upper limit of 70 micrograms per cubic meter prompt an intelligent response from the WiFi module, as illustrated in Figure 8. This response entails the module withholding data transmission, an energy-conservation strategy designed to optimize the system’s power usage. In contrast, data points within the defined range of 35 to 70 micrograms per cubic meter indicate satisfactory air quality. These data points are then promptly and automatically relayed to the cloud servers. The selective transmission of these data, in alignment with the established air quality standards, ensures that only the most pertinent information is communicated, reducing unnecessary data transfer while preserving energy resources. This distinctive approach underscores the system’s commitment to precision and efficiency, making it adept at conserving power and delivering real-time, actionable air quality information to the cloud. By adhering to the designated PM2.5 thresholds, this intelligent system strikes an optimal balance between energy preservation and accurate air quality reporting, contributing to a more sustainable and eco-friendly deployment.

Figure 12b visualizes PM10 data to provide a compelling insight into the air quality monitoring system’s responsiveness to varying pollution levels. The dataset, monitored by the PM10 sensor, is especially critical in identifying particulate matter levels that exceed the WHO-defined threshold of 70 micrograms per cubic meter. These elevated levels can indicate deteriorating air quality and potentially harmful environmental conditions. One of the remarkable features of this visualization is the display of an “empty” PM10 window, symbolizing instances when the particulate matter levels fall below the critical threshold of 70 micrograms per cubic meter. This deliberate absence serves as a powerful visual cue, indicating that, during such periods, the air quality is deemed acceptable and within the defined safety standards. Conversely, when the PM10 data surpass the 70 microgram per cubic meter threshold, they instantaneously populate the PM10 window with real-time readings. This instantaneous display signifies a critical event when the air quality is of concern, warranting immediate attention and action. By presenting PM10 data in this manner, the visualization intuitively guides users’ attention to critical variations in the air quality, ensuring that instances of elevated particulate matter levels above the defined threshold are instantly recognized. Such clear and visual reporting empowers individuals and authorities to take prompt measures to address air quality issues, reinforcing the system’s commitment to enhancing environmental safety and public health.

**Figure 13:**
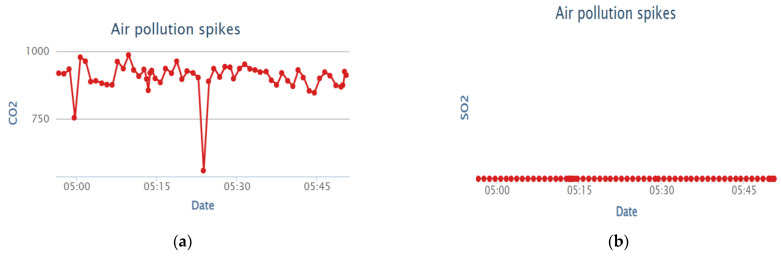

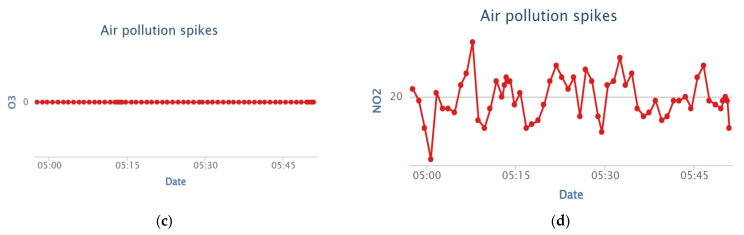
(**a**) CO2 data on the server. (**b**) SO2 data on the server. (**c**) Ozone data from the server. (**d**) NO2 data from the server.

Figure 13a provides valuable insights into the system’s capacity to monitor carbon dioxide levels, a key indicator of indoor air quality. With a defined threshold set at 1000 micrograms per cubic meter (µg/m^3^), this visualization is crucial for assessing environmental conditions and air safety. The laboratory data showcased in this visualization notably reveal that the CO2 levels consistently remain below the defined threshold of 1000 µg/m^3^. This can be attributed to the laboratory’s controlled environment, which includes air conditioning (AC) and air purification systems. These controlled conditions effectively maintain clean and healthy air quality, safeguarding against harmful concentrations of CO2. However, it is essential to recognize that during real-world deployment, the monitoring system will operate in environments that may lack the luxuries of air conditioning and air purification. The CO2 sensor’s data will become even more critical in such settings. The data will be actively transmitted only when the CO2 levels exceed the critical threshold of 1000 µg/m^3^. This transmission strategy optimizes energy usage and network resources, ensuring that information is shared when it matters most—in the presence of elevated CO2 concentrations that could potentially impact human health and comfort. This visualization thus underscores the adaptability of the air quality monitoring system, demonstrating its ability to differentiate between safe, well-controlled environments and those where the threat of elevated CO2 levels looms. It empowers users with the timely information necessary to take appropriate actions, safeguarding against potential health risks and optimizing indoor air quality.

In Figure 13b, the SO2 sensor data visualization is a testament to the precision and reliability of the monitoring system. In the controlled environment of the laboratory, the SO2 sensor consistently registers a reading of 7.3 micrograms per cubic meter (µg/m^3^). This level is significantly below the threshold defined in Table 1, which underscores the absence of this particular pollutant in the laboratory setting. The consistency in the SO2 sensor data is not surprising, given that the laboratory is a clean and controlled space, free from the emissions and pollutants typically associated with transportation-related activities. In such an environment, the absence of SO2 is expected, and the sensor readings reaffirm this expectation. However, the significance of this visualization lies in its implications for transportation-related pollution analyses. While the laboratory environment remains unpolluted by SO2, the monitoring system’s capability to measure even trace amounts of this pollutant is crucial for real-world applications. In urban and industrial areas where transportation activities are prevalent, SO2 emissions from sources such as vehicle exhausts can pose significant health and environmental risks. Although well below the defined threshold, the stable reading of 7.3 µg/m^3^ serves as a baseline for SO2 levels in clean environments and highlights the system’s sensitivity to even minimal concentrations of this pollutant. When deployed in transportation networks or areas with potential SO2 emissions, the system can detect and report any increases above this baseline, providing essential data for pollution analyses and enabling timely responses to maintain air quality and public health. In essence, this visualization showcases the system’s accuracy and consistency and emphasizes its critical role in monitoring pollutants such as SO2 in real-world scenarios. It is a testament to the system’s adaptability and the importance of collecting comprehensive air quality data to benefit public health and environmental well-being.

Figure 13c gives the data representation from the MQ131 sensor, tasked with capturing ozone (O3) levels, and offers a unique perspective on the capabilities and potential of the monitoring system. The data in the controlled laboratory experiment show a constant value of 0. This result is entirely predictable, given that the study was conducted indoors, and indoor settings are typically devoid of ozone exposure, resulting in a stable and unchanging reading. The MQ131 sensor’s constant reading of 0 in the laboratory environment serves as a reminder of the controlled conditions of the experiment. Its future role in outdoor deployments promises to provide critical data for ozone concentration, which will contribute significantly to our understanding of air quality and the impact of ozone on public health and the environment. This shift from a static indoor reading to real-world outdoor measurements underscores the system’s adaptability and potential to contribute to a healthier, more informed society.

Lastly, Figure 13d offers a valuable glimpse into the dynamic nature of the NO2 sensor’s measurements. As observed in the laboratory experiment, the data fluctuate, exhibiting variations in response to the environmental conditions. These fluctuations are completely anticipated since the nitrogen dioxide (NO2) levels in the atmosphere can be affected by various factors, including traffic emissions, industrial operations, and meteorological conditions. In the laboratory environment, the NO2 sensor’s data remain consistently below the threshold defined in Table 1, which is 80 micrograms per cubic meter. This outcome is mainly due to the controlled indoor setting, which is not subjected to the various pollution sources and atmospheric dynamics encountered in outdoor environments. However, it is essential to note that the NO2 sensor is specifically designed for real-world outdoor deployments, where it will fulfill its vital role in monitoring nitrogen dioxide levels. The sensor will transmit data only when the measured NO2 levels exceed the predefined threshold of 80 micrograms per cubic meter. This threshold is under the World Health Organization’s (WHO) guidelines for NO2 concentrations in outdoor air. Nitrogen dioxide is a significant air pollutant, primarily arising from combustion processes in vehicles, power plants, and industrial facilities. Elevated levels of NO2 in the atmosphere can adversely affect human health, particularly the respiratory system. Monitoring and regulating NO2 levels are essential for assessing air quality, protecting public health, and implementing effective pollution control measures. The NO2 sensor’s capability to transmit data selectively, focusing on instances when pollution levels surpass the established threshold, is a practical approach to data management. It ensures that the system efficiently utilizes resources and provides the most relevant information for environmental and health assessments. As a result, the NO2 sensor’s data will serve as a critical tool in understanding and addressing the impact of nitrogen dioxide on air quality, offering valuable insights and contributing to the overall mission of creating healthier and more sustainable urban environments.

### 4.2. Analysis of Spikes

**Figure 14:**
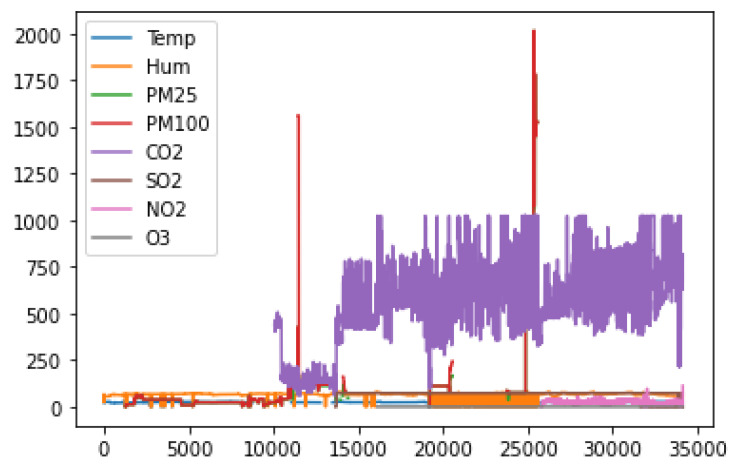
Data from all sensors.

This research scrutinizes data from each sensor employing the designated algorithm. Presented below are the resultant figures of this analysis. Figure 14 vividly illustrates all the sensors, each represented by distinct colors in the dataset.

Figure 14 offers a comprehensive view of the extensive dataset acquired from various sensors, all meticulously corrected by the prototype developed within the confines of the laboratory at Seoul National University. This rich dataset encompasses a staggering total of approximately 35,000 rows of meticulously collected data. An essential point to consider is that the data points within this dataset do not synchronize with the same starting time due to the unique initiation process of the system. This initiation process commenced precisely when the very first sensor, the temperature and humidity sensor, initiated its data collection. To visually represent these datasets, temperature readings are labeled as “Temp” and distinguished by a unique shade of blue, while humidity values are denoted as “Hum” and vividly depicted in a striking orange hue. This figure serves as a window into the intricate world of the data collected and is instrumental in understanding the dynamic environmental conditions observed throughout the data acquisition process.

Moving on to the pollution-related sensors, Figure 14 showcases PM2.5 and PM10 data in green and red, respectively. Notably, their values align closely, and further details regarding these pollutants will be presented in the subsequent figures, which will delve into each sensor’s performance in detail. Notably, the PM2.5 sensor was added shortly after the system’s inception, so its data do not initiate at index 0. The presence of peaks in both the PM2.5 and PM10 data suggests the occurrence of spikes, emphasizing the necessity for the system to detect these spikes using Chart 1.

The purple-colored data points represent readings from the CO2 sensor, with the graph revealing that these measurements were initiated after the installation of the previously explained sensors. Meanwhile, the SO2 sensor’s data, depicted in brown, seem relatively constant. The gray-colored data signify ozone levels, which consistently register at zero. Finally, the pink data points correspond to NO2 measurements, representing the most recent addition to the sensor array. The subsequent section will expound on the discussion of each sensor’s results.

This section is an illuminating showcase of the outcomes achieved through the meticulously designed prototype, complemented by the ingenious algorithm at the heart of the research. The primary focus of this section has been detecting pollution spikes within the extensive dataset amassed through the sensor network. As a result, we have gained invaluable insights into the air quality dynamics within transportation networks. Furthermore, this section has unveiled the results for each sensor, showcasing their respective performance as observed on the cloud server. These results offer a glimpse into the real-world behavior of these sensors as they capture data from the environment, providing a rich source of information for subsequent analyses and decision making. In essence, this section represents the culmination of a multifaceted research endeavor, combining cutting-edge technology, mathematical rigor, and practical implementation to achieve a holistic understanding of air pollution in transportation networks. The dataset presented here is a testament to the comprehensive nature of this study, encompassing a wide array of environmental parameters crucial for assessing air quality and its impact on public health. As we embark on the forthcoming section, we will delve even deeper into the interpretation and implications of these results. This analytical journey will unlock the hidden meanings within the data, extract actionable insights, and explore the ramifications of these findings for urban planning, public health, and environmental sustainability.

## 5. Discussion

**Figure 15:**
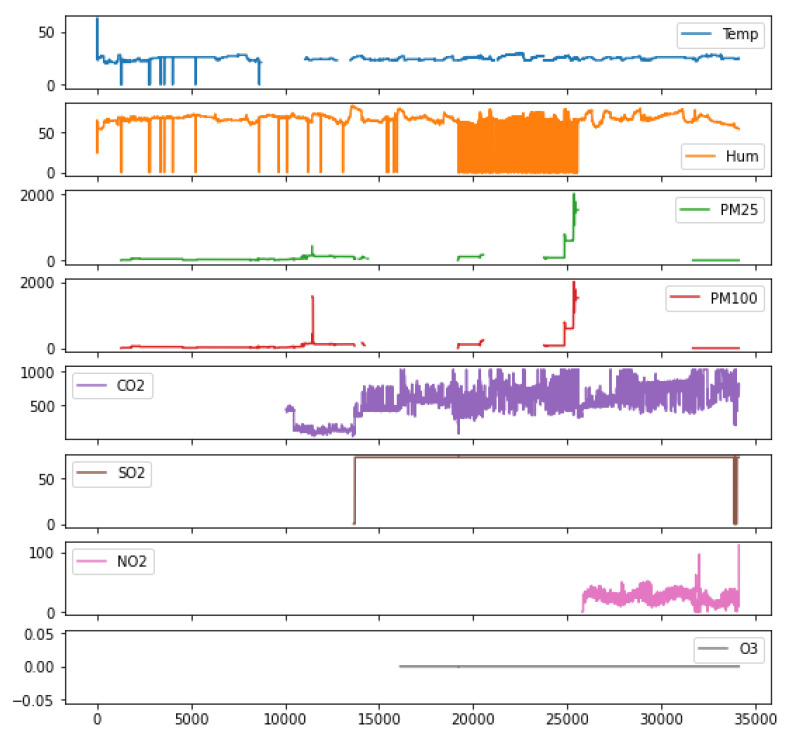
Data manipulation from each sensor.

This section delves into a comprehensive discussion of the results previously presented. It entails thoroughly examining and explaining the outcomes obtained from each sensor. The significance of the data hinges on the algorithm’s design and the specific thresholds delineated in Table 2 for each sensor. These pivotal results are graphically depicted in Figure 15.

In Figure 15, the analysis of the temperature sensor data reveals intriguing insights. During this experiment, the direct temperature values, prominently indicated within the temperature portion of the dataset, were recorded exclusively during intervals when the sensor refrained from transmitting data to the cloud. This intriguing behavior stemmed from the ambient temperature being consistently maintained below the sensor’s predefined threshold of 22 °C. Consequently, the temperature remained relatively stable throughout the experiment. On the contrary, the humidity data exhibited a stark contrast, maintaining a remarkable consistency and showing negligible fluctuations. However, it is important to note that discrete data points might emerge by marginally lowering the system’s defined threshold, shedding light on the subtle nuances of environmental conditions.

The data stemming from the PM2.5 sensor, capable of capturing particles with diameters of 2.5 μm, and the PM10 sensor, which accommodates larger 10-micrometer particles, exhibit a discrete pattern. When these particulate matter levels fall below their predefined thresholds, they are incorporated into the dataset, offering a distinct visualization of air quality. With its stable air conditioning (AC) system, the laboratory environment facilitated the successful capture and transmission of data from the CO2 sensor. However, it is imperative to define appropriate thresholds for this sensor since it sporadically detects signal spikes, a topic of forthcoming discussion in the subsequent section.

The performance of the SO2 sensor is equally fascinating. The data originating from this sensor remained constant throughout the experiment, consistently measured well below the threshold of 50 micrograms per cubic meter, which is equivalent to 0.05 parts per million (ppm). To visualize these minuscule values more effectively on the graph, they have been ingeniously scaled by a factor of 1000. In the controlled laboratory setting, the SO2 sensor consistently registers a low value of 0.0073 ppm, highlighting its precision and reliability in monitoring this pollutant, which, while largely absent within the laboratory, plays a pivotal role in transportation-related pollution analyses. These intriguing findings set the stage for deeper discussions and explorations in the subsequent sections of our research.

The data obtained from the NO2 sensor indicate that the expected values are typically below 80 micrograms per cubic meter, which aligns with the threshold defined in Table 1. However, it is important to note that the NO2 sensor installed in the laboratory setting demonstrates some variability in its readings over time. This variability is evident in the fluctuations observed in the data. Upon closer examination, it becomes apparent that the NO2 sensor readings occasionally exhibit spikes, as illustrated in Figure 15. These spikes represent instances where the NO2 levels temporarily rise above the anticipated range, reaching values around 100 micrograms per cubic meter. This phenomenon underscores the sensor’s capability to detect short-lived pollution events or localized sources of NO2 emissions. These occasional spikes provide valuable insight into the dynamic nature of air quality, even in a controlled laboratory environment. This serves as a reminder that many factors, including environmental conditions, human activities, and industrial processes, influence pollution levels. The NO2 sensor’s capacity to record these variations and spikes establishes it as a vital component in our air quality monitoring system, mainly when utilized in real-world environments characterized by a broader range of pollution sources that can be highly unpredictable. The subsequent sections of our research will delve deeper into interpreting such spikes and their implications for monitoring and managing air quality in transportation networks.

The data derived from the ozone sensor within the laboratory environment consistently register at a value of zero. This outcome is attributed to the specific conditions within the room, characterized by continuous air conditioning (AC) and limited exposure to outdoor pollutants. The indoor atmosphere, as a controlled environment, starkly contrasts the conditions in which this system is intended to operate when deployed outdoors. In outdoor settings, environmental conditions tend to be more dynamic and pollutant-laden. The ozone levels in these settings are anticipated to be substantially higher than the constant zero value observed in the laboratory. Outdoor environments inherently contain many pollutants stemming from various sources, including vehicular emissions, industrial activities, and natural factors. The exhaust emitted by vehicles, where this system is primarily designed to be deployed, is particularly significant in contributing to elevated ozone levels and other harmful pollutants. As a result, the ability to capture and analyze real ozone measurements in these dynamic outdoor conditions is of paramount importance. The system is poised to provide invaluable insights into the complex interplay of pollutants and environmental factors, furthering our understanding and enhancing our ability to address air quality challenges effectively. The forthcoming sections of our research will explore the implications of these findings in greater detail.

**Figure 16:**
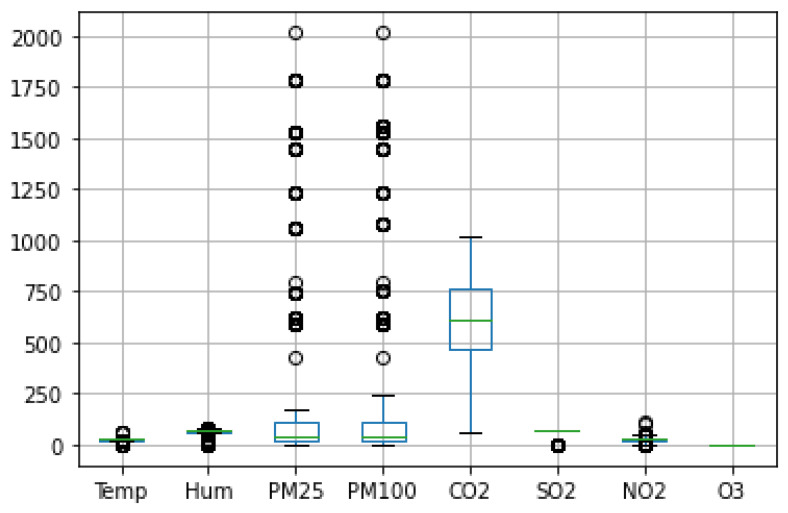
Distribution of data from all sensors.

In Figure 16, the data from various sensors are visually represented in their respective distributions based on their median values. The graph clearly illustrates that the data about different pollutants are entirely independent.

In Figure 16, the data extracted from the PM2.5 sensor paint a compelling picture, revealing a substantial presence of spikes within the dataset. The dataset contains over 2000 instances of elevated values for PM2.5, surpassing the established threshold of 35 micrograms per cubic meter, as indicated in Table 2. These thresholds serve as essential benchmarks, setting the upper limits for acceptable air quality. To put this into perspective, the World Health Organization (WHO) and other environmental regulatory bodies have defined these thresholds to safeguard public health. In ideal conditions, the air quality should not exceed these limits to prevent adverse health effects, particularly on vulnerable populations.

Hence, the abundance of spikes, each indicative of elevated PM2.5 levels, is a cause for concern.

Moreover, it is essential to recognize that these spikes in PM2.5 levels are intertwined with factors that can directly impact air quality. Transportation activities, industrial processes, and environmental conditions can all contribute to fluctuations in PM2.5 concentrations. Monitoring and addressing these spikes is paramount to mitigating the potential health risks posed by exposure to delicate particulate matter, which can infiltrate the respiratory system and lead to various health issues, including respiratory diseases, cardiovascular problems, and even premature mortality. The revelation of over 2000 elevated PM2.5 values underscores the urgent need for proactive air quality management and stringent measures to curb pollution sources. Whether through targeted policies, emissions controls, or public awareness campaigns, addressing the root causes of these spikes is essential to ensuring clean and breathable air for all. These findings serve as a potent reminder of the interconnectedness of environmental conditions and human health, emphasizing the critical role that real-time monitoring and data analyses play in safeguarding our well-being and the environment. As the paper proves, these values can harm children [25].

Based on the results obtained, it becomes evident that this paper has successfully developed the proposed system, which finds direct applicability in the vehicles discussed in Paper [39]. Furthermore, the findings of this research shed light on the considerable impact of memory consumption when utilizing this system, as demonstrated in Paper [37]. Papers [41,42,43,44,45] presented various suggestions for addressing air pollution in smart cities, and this research offers a concrete solution to these proposals, marking a significant contribution to the field.

The “Prototype for Monitoring Transportation Pollution Spikes through IoT Edge Networks” study offers valuable insights into real-time air pollution detection, particularly concerning the dynamic context of transportation. In this section, we delve into a comprehensive analysis of the study’s findings, shedding light on the behavior of the various sensors employed to measure critical environmental parameters and pollutant levels within a meticulously controlled laboratory setting.

The study’s results cast a spotlight on several key observations. Firstly, the data acquired from the temperature sensor unveil a clear correlation with data transmission. Specifically, the temperature data were recorded when the sensor was not transmitting to the cloud, coinciding with temperatures below 22 degrees Celsius. In contrast, the humidity data remained consistent throughout the experiment and did not exhibit notable fluctuations below the predefined threshold. However, the study raises the possibility that the system’s entry should be slightly adjusted; discrete data points could emerge in the humidity dataset.

Moreover, the data from the PM2.5 sensor, which measures particulate matter levels with 2.5 mm and 10 mm diameters, exhibited discrete patterns. These discreet data points were primarily observed when the sensor’s readings fell below the predefined threshold values. This underscores the sensor’s ability to discern and transmit data effectively when pollution levels surpass the specified thresholds.

Considering that the laboratory environment in which the system was situated maintained a stable air conditioning (AC) system, the data captured by the CO2 sensor remained consistent and aligned with expectations. However, it is worth noting that, on occasion, this sensor detected spikes in the signals. These occasional spikes raise intriguing questions about the dynamics of indoor air quality, hinting at the need for further exploration to comprehend these phenomena better.

The study also examined the data from the SO2 sensor, which exhibited a remarkable degree of stability. The data consistently registered low levels of SO2, well below the defined threshold of 50 micrograms per cubic meter, which is equivalent to 0.05 parts per million (ppm). To enhance the visualization of these small values on the graph, they were scaled by a factor of 1000. The laboratory environment was notably free of this pollutant, which is crucial for a transportation-related pollution analysis.

In contrast, the ozone (O3) sensor readings remained at zero within the indoor laboratory setting. However, it is imperative to acknowledge that these values are expected to significantly deviate from this consistent zero level when deployed in outdoor environments. Outdoor settings inherently contain a broader spectrum of pollutants, making the accurate measurement and monitoring of ozone levels a pivotal aspect of the study’s real-world applicability, particularly in transportation.

Last, the study scrutinized data obtained from the NO2 sensor. It was determined that the expected values should generally fall below 80 micrograms per cubic meter, adhering to the threshold outlined in Table 2. However, as the sensor was also situated within the laboratory, it exhibited variations in readings over time. Notably, the data displayed occasional spikes, as shown in Figure 15, with readings occasionally surging to around 100 micrograms per cubic meter. These intermittent spikes underscore the sensor’s potential to detect and respond to short-lived pollution events or localized sources of NO2 emissions, an invaluable attribute for monitoring air quality in dynamic transportation scenarios.

In conclusion, the “Prototype for Monitoring Transportation Pollution Spikes through IoT Edge Networks” study offers a wealth of insights into real-time air pollution detection. These findings collectively advance our understanding of this critical field, particularly within the context of transportation. The results provide valuable implications for health and environmental management, underscoring the significance of proactive and precise air quality monitoring to ensure the well-being of communities and the sustainable management of our shared environment.

## 6. Conclusions

In summary, the paper titled “Prototype for Monitoring Transportation Pollution Spikes through IoT Edge Networks” stands as a significant milestone in air pollution monitoring and management, with a particular focus on the transportation sector. The research showcases an innovative approach that leverages the power of IoT edge networks and a diverse array of sensors to enable the real-time detection of air pollution spikes. Sensors for PM2.5, PM10, CO2, NO2, SO2, and O3 were used to capture the levels of these pollutants emanating from vehicles during their movement. The study’s results speak volumes about the system’s efficiency in capturing and analyzing pollutant spike data, offering valuable insights into the intricacies of environmental dynamics within the controlled confines of a laboratory setting. The results from the laboratory show that the O3 reading was at zero and the SO2 reading was unchanging but could not cause any harm. The PM 2.5, PM10, CO2, and NO2 sensors’ values all changed accordingly, with some spikes in the environment of the laboratory. These findings transcend the confines of the laboratory and hold immense significance for public health and urban environmental management. The ability to promptly identify pollution spikes in real time equips us with the means to initiate swift interventions and preventive measures, ultimately safeguarding the well-being of urban populations. This research lays a strong foundation for advancing air quality monitoring in the context of transportation, promising to contribute to creating healthier and more sustainable urban environments. The research is poised to take a significant step forward by shifting its focus to encompass a broader analysis of data collected from various locations where the system is deployed. This expansion will usher in new challenges, particularly regarding data management and security. In this regard, integrating blockchain technology will play a pivotal role in addressing these challenges and ensuring the integrity and traceability of the data collected. Additionally, adopting machine learning models is set to enhance the system’s predictive capabilities, allowing for a more proactive approach to pollution management. As the research embarks on this next phase, it holds the potential to revolutionize how we perceive and address air quality issues in transportation, fostering a cleaner and healthier future for urban dwellers.

## Figures and Tables

**Figure 2:**
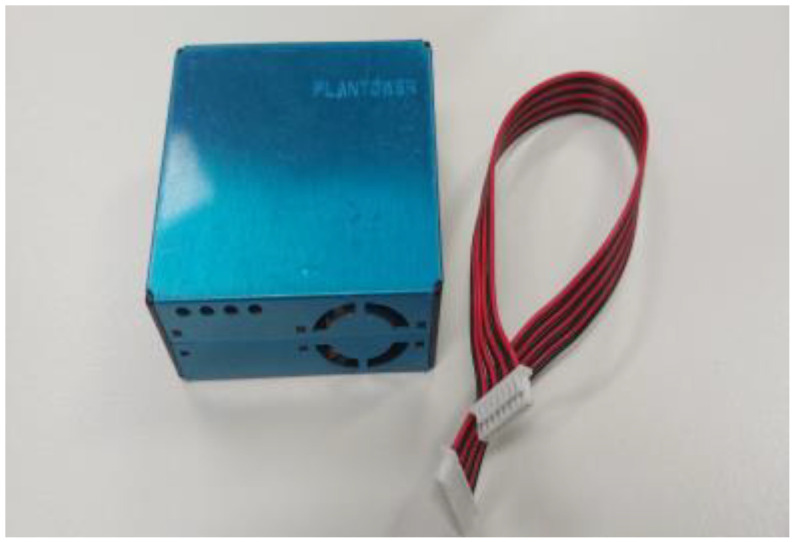
PM2.5 and PM10 sensor.

**Figure 3:**
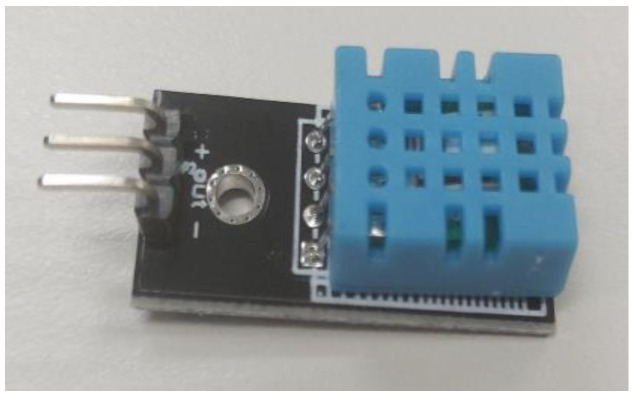
Temperature and humidity sensor.

**Figure 4:**
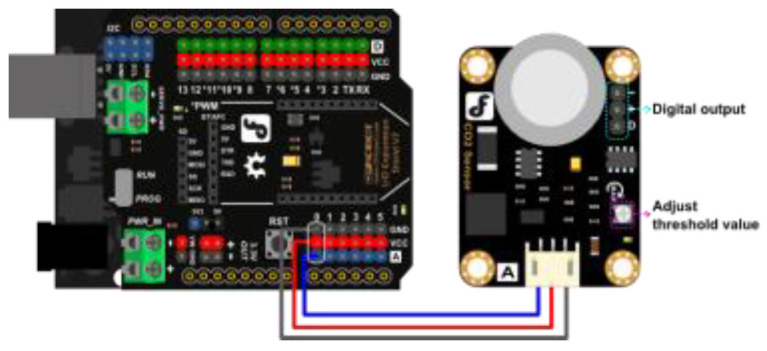
CO2 sensor.

**Figure 5:**
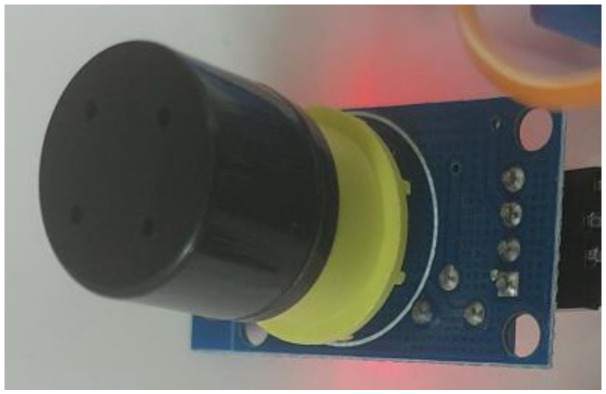
MQ131 sensor for ozone.

**Figure 6:**
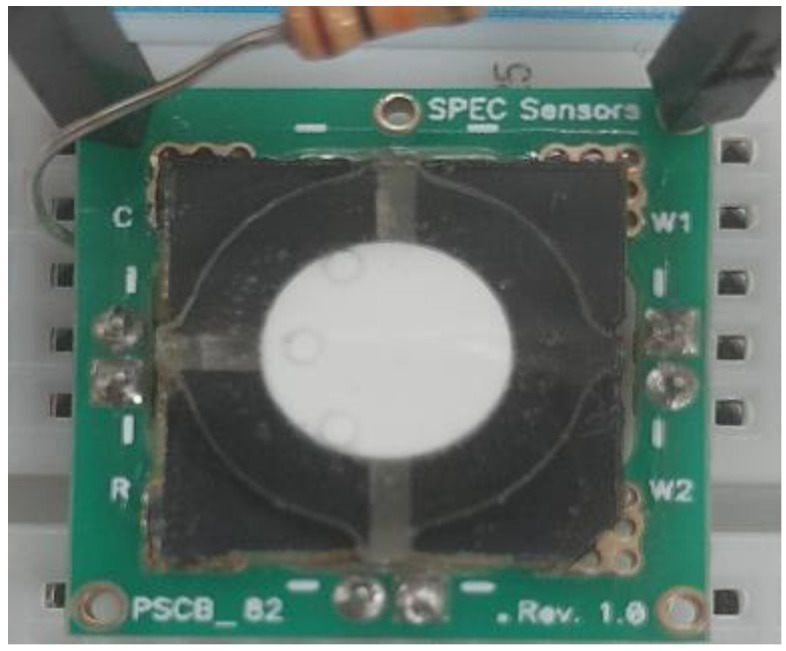
SO2 sensor.

**Figure 7:**
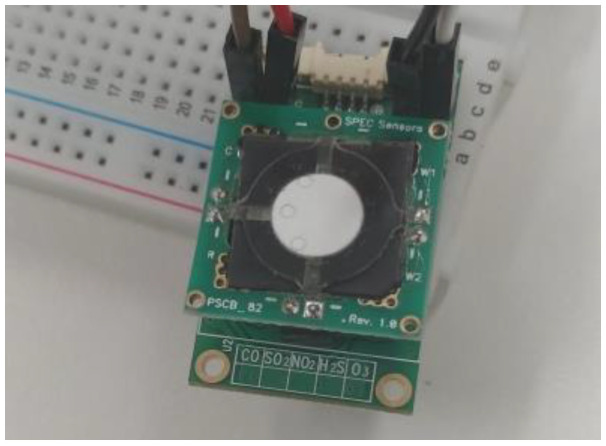
NO2 sensor.

**Table 1:** Comparison of the existing systems and the current system.

Existing Work	Current Research
Introduces a novel approach for designing a cost-effective and real-time air pollution monitoring system using edge computing and IoT technologies [34].	Implements the novel design by applying it in urban areas with vehicles.
Using multisensory and weather data, developing an AI model, to measure traffic-related air pollution [34].	Only a few pollutants were considered; the current research assesses six pollutants. In addition, air pollution was measured in general, yet this research will consider the pollutants from each vehicle.
Development of portable IoT air quality monitoring devices for vehicles for intelligent transportation systems [34].	These systems have been developed and installed on cars. However, they are installed on the top of a vehicle and can measure air pollution for the environment in general without considering the pollutants from the vehicle’s exhaust. Also, they capture pollution without considering spikes of it that are to be reported as remarkable, as is carried out in the current research.
Development of systems that are only specific to some pollutants [34].	The current research developed a system that comprises six dangerous pollutants.
Development of a framework for a smart city for air pollution monitoring [34].	The current research has designed a prototype based on the framework proposed for smart city air pollution monitoring, with the addition of the capability to record and monitor the exhaust of air pollution from vehicles.

**Table 2:** The minimum concentration of air pollutants defined by WHO.

Air Pollutant Name	Minimum Concentration (µg/m^3^)
Particulate Matter (2.5)	35
Particulate Matter (10)	70
Carbone dioxide (CO2)	1000
Nitrate of Oxide	80
Sulfur of Oxide	50
Ozone	120

## Data Availability

The dataset used in this research can be downloaded from https://thingspeak.com/channels/2178832. Some graphs presented in this paper can be found on this link.
